# Recent Advances in Photo−Activated Chemical Sensors

**DOI:** 10.3390/s22239228

**Published:** 2022-11-27

**Authors:** Dong Hyun Lee, Hocheon Yoo

**Affiliations:** Department of Electronic Engineering, Gachon University, 1342 Seongnam−daero, Seongnam 13120, Republic of Korea

**Keywords:** photo−activated gas detectors, ultraviolet, visible light, nitric oxide, nitric dioxide, formaldehyde, ammonia

## Abstract

Gas detectors have attracted considerable attention for monitoring harmful gases and air pollution because of industry development and the ongoing interest in human health. On the other hand, conventional high−temperature gas detectors are unsuitable for safely detecting harmful gases at high activation temperatures. Photo−activated gas detectors improve gas sensing performance at room temperature and enable low−power operation. This review presents a timely overview of photo−activated gas detectors that use illuminated light instead of thermal energy. Illuminated light assists in gas detection and is classified as visible or ultraviolet light. The research on photo−activated gas detectors is organized according to the type of gas that can be intensively detected. In addition, a development strategy for advancing photo−activated gas detectors is discussed.

## 1. Introduction

**Figure 1:**
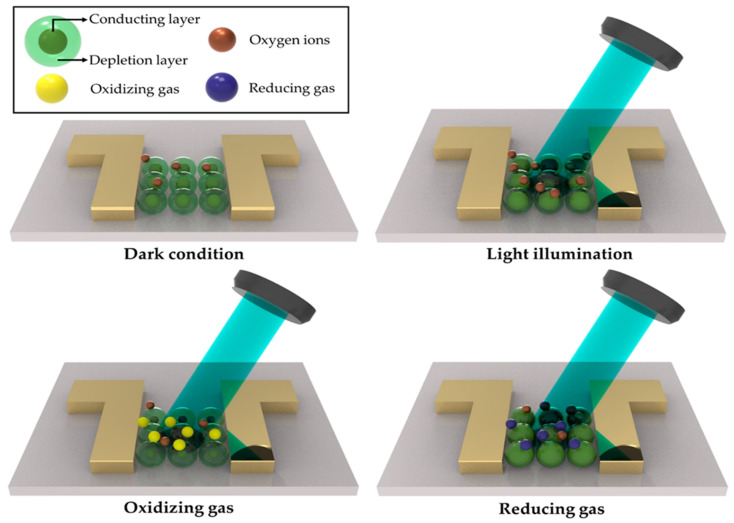
Surface statement of n−type metal oxide semiconductor−based photo−activated gas sensor under oxidizing gas and reducing gas.

Gas detectors have attracted enormous attention because they can detect the emergence of a gas in areas of development for particular safety systems. To be specific, gas detectors offer rapid detection for early response to tremendous accidents with flammable [1,2,3,4,5,6,7,8,9], combustible gases [10,11,12,13,14,15], as well as bio−hazardous gases [16,17,18,19,20,21,22,23,24,25]. These devices are widely used in industry for continuously monitoring gas leakages [26,27,28,29] or manufacturing processes [30,31,32,33]. Gas detectors can be classified according to their operation principles: electrochemical devices [34,35,36,37], photoionization−based types [38,39,40,41], ultrasonic detectors [42,43,44,45], and semiconductor−based devices [46,47,48,49]. Among the various detector types, semiconductor−based devices allow rapid gas detection because the electrical resistance changes when it comes into contact with the target gas. Semiconductor−based gas detectors have been developed, and a variety of detectors have been reported using semiconducting materials, such as metal oxides [50,51,52,53,54], carbon nanotubes [55,56,57,58], two−dimensional materials, including graphene [59,60,61,62] and transition metal dichalcogenide [63,64,65,66], organic materials [67,68,69,70], and perovskites [71,72,73,74]. On the other hand, these detectors suffer cross−sensitivity with other gases; high reactivity with gases other than the target gas reduces selectivity [75,76,77]. Furthermore, the demand for faster recovery rates [78,79,80] and improved responsiveness in detecting extremely small amounts of gas [81,82,83,84] is driving the continuous development of these detectors. As an emerging approach, photo−activated or photo−assisted gas detection over diverse materials and devices has been proposed. By irradiating with light of a specific wavelength during the gas detection operation, this approach (1) enhances the photoreaction speed and recovery speed, (2) secures gas selectivity, and (3) increases the gas reactivity. The mechanism of gas sensors is different for each sensor device. Electron−hole pairs are generated by the interaction between the light irradiated on the photo−activated gas sensor and the surface of the gas−sensitive material. Photo−generated charge carriers interact with the oxygen ions of the gas−sensitive material, resulting in a change in conductivity. Figure 1 shows the surface statement of an n−type metal oxide semiconductor−based photo−activated gas sensor by injecting oxidizing gas and reducing gas.

In this context, this review comprehensively revisits recent advances in photo−activated or photo−assisted gas detectors, emphasizing their illuminated light wavelengths and target gases. This paper reviews the recent developments in photo−activated gas detectors, classified according to the type of light source and target gas. The light sources for photo−activated gas detectors discussed in this review are ultraviolet and visible light. In addition, studies of photo−activated gas detectors with high selectivity for nitric oxide, nitric dioxide, formaldehyde, and ammonia gases are summarized. Finally, technology development strategies for photo−activated gas detectors are offered. 

## 2. UV−Activated Gas Sensors

### 2.1. UV−Activated Nitric Oxide Gas Sensors

**Figure 2:**
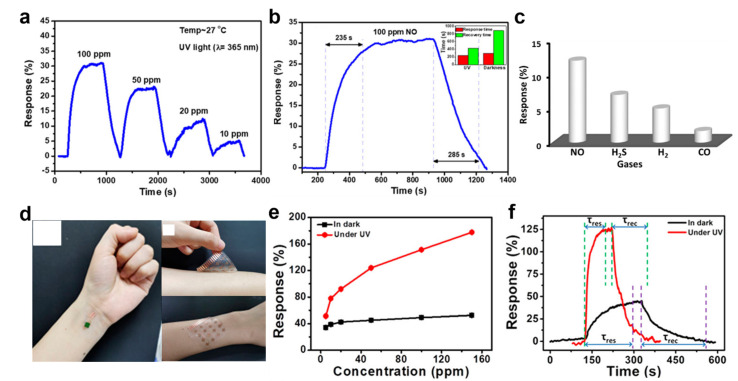
(**a**) Response of the TiO_2_@NGQDs sensor under UV irradiated; (**b**) response and recovery times of TiO_2_@NGQD sensors with UV−on and UV−off; (**c**) selectivity characteristics of the TiO_2_@NGQDs sensor (adapted from [85] with permission from the American Chemical Society); (**d**) image of the flexible and wearable TiO_2_@NGQDs sensor with human arm and wrist; (**e**) response of the Cu−TCA/TiNCs sensor ppb with UV−on and UV−off; (**f**) response and recovery time of the Cu−TCA/TiNCs sensor (adapted from [86] with permission from the American Chemical Society).

As a remarkable example of UV−activated nitric oxide gas detection, in 2020, Murali et al. presented NO gas detection using a heterojunction structure based on nitrogen−doped graphene quantum dots/titanium dioxide (TiO_2_) named NGQDs [85]. The {001} facet form with unsaturated coordination atoms and dangling bonds led to more absorbed oxygen−related gases on the TiO_2_ surface, inducing high responsivity on the gas sensor platform. NGQDs were fabricated by doping graphene quantum dots (GQDs) with nitrogen atoms, which increased the gas−detecting performance of the heterostructure TiO_2_ gas sensor by increasing the charge carriers and defects. Figure 2a shows the NO gas sensing characteristics of the TiO_2_@NGQDs sensor irradiated with UV light (λ = 365 nm). The response to 10−100 ppm NO gas was investigated. A higher concentration of injected NO gas increased the resistance of the TiO_2_@NGQDs sensor. The measured response time and recovery time were 235 and 285 s, respectively (Figure 2b); they were 63 s and 605 s faster than under dark conditions. When irradiated with UV light, the response speed was improved due to the more active absorption and desorption of oxygen species on the TiO_2_ surface than under dark conditions. Furthermore, the selectivity of the TiO_2_@NGQDs sensor was investigated (Figure 2c). The TiO_2_@NGQDs detected 100 ppm of NO, H_2_S, H_2_, and CO at room temperature. On the other hand, the response to NO gas was up to seven times higher than other gases. When n−type TiO_2_ and p−type NGQDs have heterostructures, the excess electrons of TiO_2_ move to NGQDs due to the relatively low bandgap of TiO_2_. The NO gas injected into the TiO_2_@NGQDs sensor interacts with surface oxygen ions and electrons in TiO_2_, which reduces the carrier concentration of TiO_2_. On the other hand, when the TiO_2_@NGQDs sensor is irradiated with UV light, it interacts with the generated photoelectrons and oxygen on the surface to produce oxygen ions. The additionally generated UV light−induced oxygen ion species interact with the larger number of electrons in TiO_2_.

As another example, a p−n heterostructure was used to detect NO gas. He et al. represented a UV−activated NO gas sensor operable at room temperature using Cu−TCA (H3TCA = tricarboxytriphenylamine) and TiO_2_ nanochannels (TiNCs) as a p−n heterostructure [86]. The Cu−TCA porous structure, due to the metal−organic framework (MOF), allows only the NO gas contained in the mixed gas to be adsorbed, providing selectivity for NO gas. In addition, the porous structure has many active sites for absorbing more NO molecules. The TiNCs as photocatalytic materials showed improved NO gas sensing characteristics owing to their high surface−to−volume ratio and stable chemical characteristics. Figure 2d presents an image of a flexible Cu−TCA/TiNCs sensor attached to human skin. Polydimethylsiloxane (PDMS) was used as a substrate for the Cu−TCA/TiNCs sensor, and the S−shaped Cu electrode prevented damage due to fatigue deformation. The performance of the Cu−TCA/TiNCs sensor was improved when irradiated with UV light (λ = 365 nm). Figure 2e shows the NO gas response of the Cu−TCA/TiNCs sensor in dark and UV light−irradiated conditions with a range of 5–200 ppm NO gas injected. The NO gas response of the Cu−TCA/TiNCs sensor with UV light irradiation was increased from 1.5−fold to 3.4−fold compared with the dark condition. The UV irradiation effect reduced the response time of the Cu−TCA/TiNCs sensor owing to the large carrier concentration and active sites. Furthermore, the recovery time decreased owing to the accelerated activation of the surface to return to its initial state. Figure 2f shows the response speed of the Cu−TCA/TiNCs sensor. The response and recovery time were decreased by 101.2 s and 106 s compared to the dark conditions. The Cu−TCA/TiNCs sensor detected NO molecules due to a change in resistance by reactive oxygen species. The NO molecules capture electrons in the Cu−TCA/TiNC conduction band and interact with oxygen ions on the surface to form NO ions and N_2_ ions. As a result, the NO and N_2_ ions adsorbed on the surface increase the resistance of Cu−TCA/TiNC. On the other hand, UV light increases the response of the Cu−TCA/TiNCs sensor to NO gas. The electron–hole pairs generated by UV light produce many oxygen ions that interact more with the NO molecules in the dark. As a result, the width of the depletion layer was larger than in the dark, resulting in high resistance. 

### 2.2. UV−Activated Nitric Dioxide Gas Sensors

**Figure 3:**
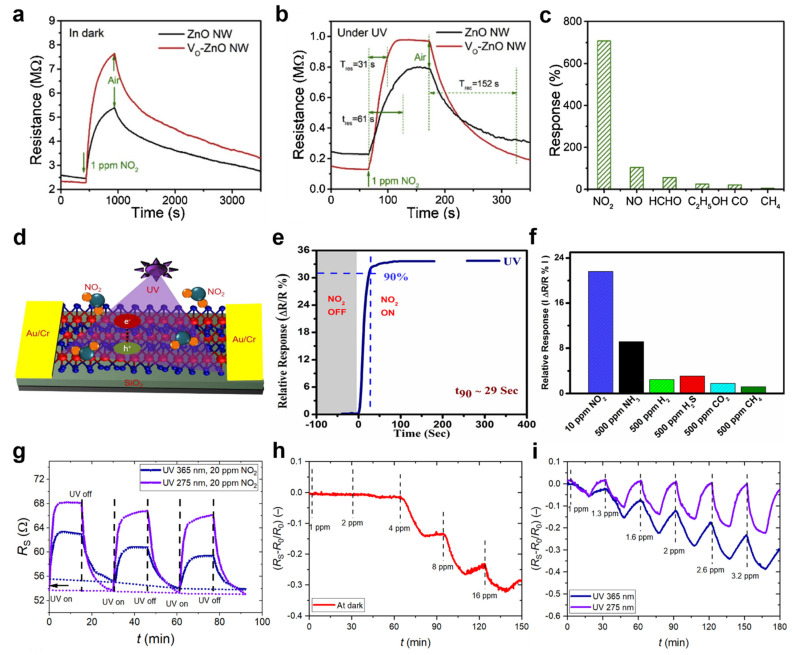
Resistance changes in the ZnO NW sensor and the Vo−ZnO NW sensor (**a**) under dark (**b**) under UV irradiation; (**c**) gas selectivity characteristics of the Vo−ZnO NW sensor (adapted from [87] with permission from the Elsevier B.V.); (**d**) schematic diagram of the MoS_2_−based gas sensor; (**e**) response time of the MoS_2_−based gas sensor under UV irradiation; (**f**) selectivity histogram of the MoS_2_−based gas sensor (adapted from [88] with permission from the American Chemical Society); (**g**) response of the CNN sensor with UV lights irradiation (λ = 365, 275 nm); (**h**) response of the CNN sensor under the dark condition; (**i**) response of the CNN sensor under UV irradiation (adapted from [89] with permission from the Elsevier B.V.).

As a representative UV−activated nitric dioxide gas detection study, in 2019, Wang et al. reported a nitric dioxide (NO_2_) gas sensor with improved sensing performance by treating ZnO nanowires (NW) with NaBH_4_ [87]. The oxygen vacancies (V_O_) on the surface of the ZnO nanowires treated hydrothermally with NaBH_4_ increase the chemisorption of oxygen species and NO_2_ molecules. In addition, the UV irradiation effect generates many oxygen ions and promotes the formation of NO_3_^−^, which effectively improves the detection performance of NO_2_ gas. Figure 3a,b show the changes in resistance of the ZnO nanowire sensor and the Vo−ZnO NW sensor with the NaBH_4_ treatment. Under UV (λ = 325 nm) irradiation and dark conditions, the NO_2_ gas sensors detected 1 ppm NO_2_ gas. The variation in resistance of the NO_2_ gas sensors depending on the initial air condition was 0.20 MΩ. Subsequently, when NO_2_ gas was injected into the NO_2_ gas sensors in the dark, the resistance of the Vo−ZnO NW was increased by approximately 2.25 MΩ compared to the ZnO NW. In other words, Vo−ZnO NW adsorbs many NO_2_ molecules due to the increased surface defects, resulting in higher changes in resistance. Under UV irradiation, the NO_2_ gas sensors showed resistance changes of 0.1 MΩ in air and 0.17 MΩ in NO_2_, respectively. UV irradiation enhanced the response and recovery time of the Vo−ZnO NW sensor. The response and recovery times of the Vo−ZnO NW sensor in UV irradiation conditions were 31 s and 144 s, respectively. The response and recovery times were reduced by 30 s compared to the dark conditions. Figure 3c shows the various gas sensing performances of the Vo−ZnO NW sensor under UV irradiation conditions. The response of NO_2_ gas was 700%, which has high gas sensing selectivity characteristics compared to other gases. In air, photoelectrons generated by UV irradiation on the Vo−ZnO NW sensor combined with oxygen species and were trapped in V_O_ to produce oxygen species. Subsequently, NO_2_ gas was injected, and the increased oxygen species interacted with the NO_2−_ species to promote NO_3_^−^ production. As a result, the depletion width was thicker, inducing high resistance.

To fabricate the gas sensor, transition metal dichalcogenides (TMDs) were used, which have excellent electrical and optical characteristics. Kumar et al. demonstrated a MoS_2_−based NO_2_ gas detection at ambient temperature using UV light illumination [88]. MoS_2_ was grown through the CVD process, and the annealing process was performed. Figure 3d presents a schematic diagram of the MoS_2_−based gas sensor operating under UV irradiation (λ = 365 nm). The MoS_2_−based gas sensor was fabricated by growing MoS_2_ on SiO_2_/Si substrates by CVD. The Au/Cr (200 nm/5 nm) electrodes were deposited by thermal evaporation, and the widths of the electrodes were 100 and 250 μm, respectively. The response time of the MoS_2_−based gas sensor to NO_2_ gas under UV light irradiation conditions was investigated (Figure 3e). The MoS_2_−based gas sensor was injected with 100 ppm NO_2_ gas. The response time was defined as the time until the saturation of the relative response reached 90% after the NO_2_ gas was injected. The response time of the MoS_2_−based gas sensor in UV conditions was 29 s, which was approximately 42 s faster than the gas response by thermal activation (100 °C). Figure 3f shows the relative responses of the MoS_2_−based gas sensor to various gases. The relative response of the NO_2_ gas measured was approximately 21%, which has the highest selectivity compared to other gases.

A gas sensor with a high surface−to−volume ratio was achieved using one−dimensional carbon nanotubes (CNT). Drozdowska et al. used a carbon nanotube network (CNN) to fabricate NO_2_ gas sensors with a high surface−to−volume ratio [89]. The CNTs have p−type semiconductor characteristics and high sensitivity to gases owing to the large number of bonding sites on their large surface. The CNN is sensitive to UV light irradiation and improves response speed. The UV light used in this study has a wavelength of 365 nm and 275 nm, respectively, and can detect at least 1 ppm of NO_2_. Figure 3g shows the response of the CNN gas sensor to 20 ppm NO_2_ gas. The NO_2_ gas response increases with the amount of UV light irradiated to the CNN gas sensor. Furthermore, the initial resistance of the CNN gas sensor increases as the UV−on and UV−off cycles are repeated. The response of the CNN sensor to various NO_2_ gases was investigated. Figure 3h shows the NO_2_ gas response of the CNN sensor in the dark. NO_2_ gas concentrations below 4 ppm are difficult to detect in the dark. On the other hand, the CNN gas sensor irradiated with UV light improves the sensing performance to detect low concentrations of NO_2_ gas, such as 1 ppm (Figure 3i). As a result, the UV irradiation increases the NO_2_ gas response of the CNN sensor. The resistance of the CNN gas sensor increased when NO_2_ was injected, and UV light irradiation increased the resistance change. UV−generated holes interact with negatively charged ions on the CNT surface, resulting in oxygen desorption. At this point, the lowered hole concentration reduces the conductivity of the CNN sensor. Additionally, the UV wavelength means that more energy is applied to the CNN sensor, resulting in the effective desorption of gas molecules, and accordingly, a rapid recovery of the CNN sensors was achieved.

### 2.3. UV−Activated Formaldehyde Gas Sensors

**Figure 4:**
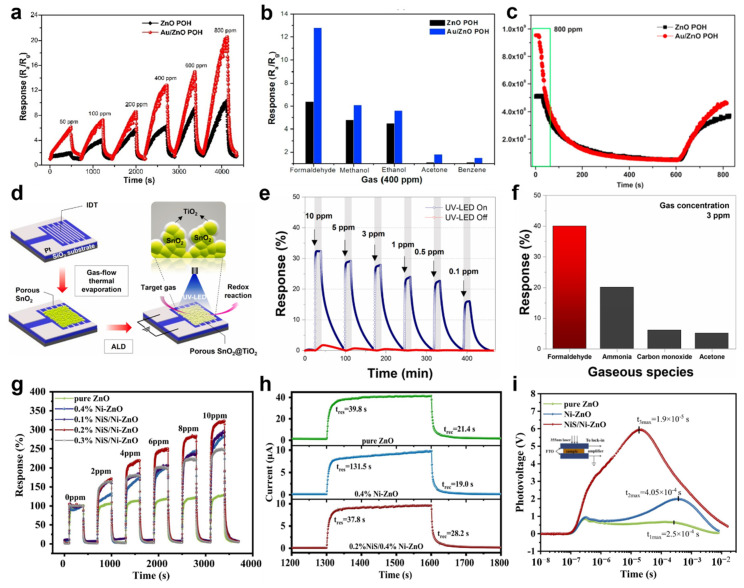
(**a**) Response of ZnO POH sensor and Au−ZnO POH sensor under UV irradiation; (**b**) response of the ZnO POH and Au−ZnO POH sensors to various gases; (**c**) actual resistance changes in the ZnO POH and Au−ZnO POH sensors (adapted from [90] with permission from the Elsevier B.V.); (**d**) schematic diagram of the SnO_2_@TiO_2_ sensor fabrication process; (**e**) response of the SnO_2_@TiO_2_ sensor in UV−on and UV−off; (**f**) selectivity characteristics of the SnO_2_@TiO_2_ sensor (adapted from [91] with permission from the Elsevier B.V.); (**g**) response of the pure ZnO, Ni−ZnO, and NiS/Ni−ZnO gas sensors to UV irradiation; (**h**) response time of the pure ZnO, Ni−ZnO, and NiS/Ni−ZnO gas sensors to HCHO gas; (**i**) spectrum of transient photovoltage (TPV) (adapted from [92] with permission from the Elsevier B.V.).

For enhancing the responsiveness of the gas sensor, a porous structure was used. Li et al. reported the formaldehyde (HCHO) gas sensor by loading Au on the porous octahedrons’ (POHs) structured ZnO surface [90]. ZnO is an n−type metal oxide that has the advantages of being low−cost and nontoxic and is used as the core material of gas sensors. On the other hand, to improve the performance of ZnO−based gas sensors, operation at high temperatures leads to high power consumption. As a solution, UV light irradiation makes ZnO−based gas detectors operate at ambient temperature, but it has a low response. The porous structure was introduced into the MOS to improve the responsivity of the gas sensor due to the high surface−to−volume ratio. Furthermore, chemical catalytic materials, such as Au, are loaded into ZnO to produce a Schottky junction, leading to improved gas sensing performance. Figure 4a shows the HCHO gas response of a ZnO POH sensor and an Au−loaded ZnO POH sensor (Au−ZnO POH). The UV light used had a wavelength of 365 nm, and 50–800 ppm HCHO gas was investigated. The Au−ZnO POH sensor has more responsiveness for all concentrations of HCHO gas than the ZnO POH sensor. In particular, the responsiveness of 800 ppm HCHO gas doubled. In this way, the Au loading effect improves its response toward HCHO molecules. In addition, the selectivity of the Au−ZnO POH sensor was investigated. Figure 4b shows the responsiveness of the Au−ZnO POH sensor to various gases. Methanol, ethanol, acetone, benzene, and formaldehyde with a concentration of 400 ppm were assessed. The responses of the Au−ZnO POH sensor to all these gases were superior to those of the ZnO POH sensor. Figure 4c shows the resistance changes in the ZnO POH and Au−ZnO POH sensors exposed to the HCHO gas. The Au loading allows more oxygen molecules to be adsorbed onto the ZnO POH surface, leading to a thicker depletion layer resulting in high resistance.

In 2021, Chang et al. produced a HCHO gas sensor based on the heterostructure of TiO_2_ and SnO_2_ to detect low HCHO gas concentrations [91]. The synergy between SnO_2_ in a porous structure and TiO_2_ as a photocatalytic material resulted in a high surface area that detected HCHO gas selectively, leading to an improved response of the gas sensor. Figure 4d shows the fabrication process of the SnO_2_@TiO_2_ gas sensor. Ti (10 nm) and Pt (150 nm) electrodes were deposited in an interdigitated shape on a SiO_2_/Si substrate. Subsequently, SnO_2_ and TiO_2_ were deposited sequentially. SnO_2_ has a nanoporous structure due to the kinetic energy of argon gas injected during the thermal evaporation process. Such a nanoporous structure can increase the response of the SnO_2_@TiO_2_ gas sensor because of its high surface−to−volume ratio and large surface area for activation. The TiO_2_ with photocatalytic material was deposited using atomic layer deposition (ALD). Figure 4e shows the HCHO gas response characteristics of the SnO_2_@TiO_2_ sensor in UV−on and UV−off. The wavelength of the irradiated UV light was 365 nm, and the concentration of the injected HCHO gas was 0.1−10 ppm. The response of the SnO_2_@TiO_2_ sensor irradiated with UV−on was increased compared to the UV−off. The increased resistance change caused by UV light irradiation allows the sensor to detect lower HCHO gas concentrations. In particular, the change in resistance to 0.1 ppm HCHO gas with UV−on increased by approximately 16% compared to UV−off. Figure 4f shows the selectivity of the SnO_2_@TiO_2_ sensor. Compared to ammonia, carbon monoxide, and acetone gas, the response to HCHO gas at 3 ppm was 40%. The electron–hole pairs generated by the UV produced reactive oxygen species on the TiO_2_ surface. The injected HCHO molecules interacted with the adsorbed oxygen ions on the TiO_2_ surface and released electrons. As a result, the released electrons accumulated in the TiO_2_ conduction band and reduced the resistance. 

To improve the responsiveness of a ZnO−based HCHO gas sensor, Yang et al. decorated nickel sulfide (NiS) nanomaterials onto Ni−doped ZnO [92]. The Ni−ZnO restrained the recombination of photo−generated electrons by UV light irradiation and increased carrier concentration. In addition, the NiS nanomaterials improved the response time of the NiS/Ni−ZnO gas sensors. Figure 4g shows the response of the HCHO gas range from 2 to 10 ppm with UV light irradiation (λ = 365 nm). The pure ZnO, Ni−ZnO, and NiS/Ni−ZnO gas sensors were compared to investigate the response of the HCHO gas. NiS decorating enhanced the response of the gas sensor. The response of the 0.4% Ni−ZnO gas sensor to 10 ppm of HCHO gas was 275%. On the other hand, the response of the 0.2% NiS/0.4% Ni−ZnO gas sensor was improved by 330%. Furthermore, the NiS improved the response time of the NiS/Ni−ZnO gas sensor. As shown in Figure 4h, the response time of the NiS/Ni−ZnO gas sensor was 37.8 s, which was significantly faster than the Ni−ZnO gas sensor with a response time of 131.5 s. Temporal photovoltage (TPV) characterizations were performed to investigate the transfer properties of photoexcited charge carriers (Figure 4i). The increased TPV response showed that the NiS/Ni−ZnO gas sensor has more photo−generated charges. In addition, the NiS/Ni−ZnO gas sensors have the fastest time to reach maximum TPV. In other words, the NiS decorated on Ni−ZnO improves the HCHO gas response time of the NiS/Ni−ZnO gas sensor by enhancing the separation and transmission efficiency of the photo−excited electrons and holes. UV light irradiation increased the change in resistance of the NiS/Ni−ZnO gas sensor. Oxygen in the air adsorbed to the surface of ZnO−based materials interacts with electrons in the ZnO conduction band promoted by UV light to generate oxygen ions. At this time, the Ni doping effect provided sites for trapping holes, enlarged oxygen ions adsorbed on the surface compared to pure ZnO. Oxygen ions adsorbed on the surface make the depletion layer of the NiS/Ni−ZnO gas sensor thicker. When HCHO gas was injected, the oxygen ions interacted with the HCHO molecule and generated electrons. The generated electrons were then transferred to the conduction band of the ZnO, increasing its conductivity. 

### 2.4. UV−Activated Ammonia Oxide Gas Sensors

**Figure 5:**
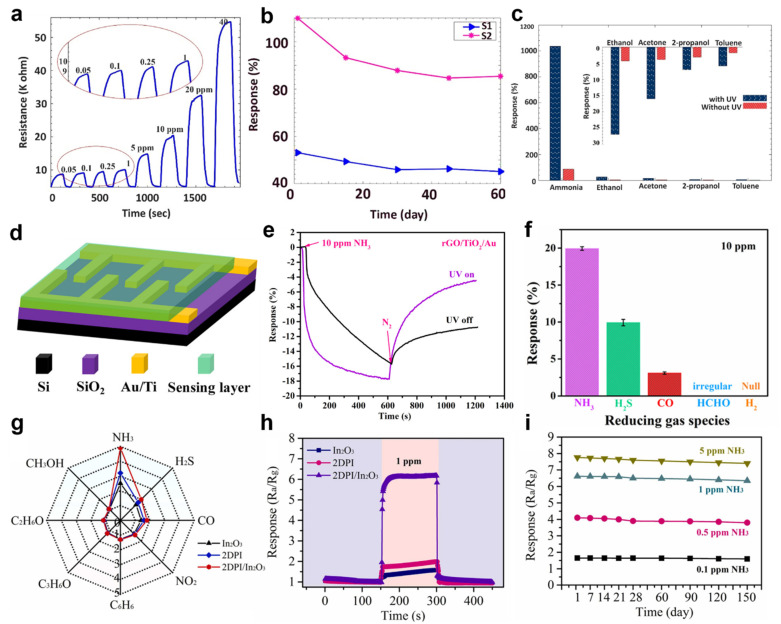
(**a**) Resistance change in the PANI/TiO_2_ core−shell sensor; (**b**) stability of the PANI/TiO_2_ core−shell sensor with UV irradiation; (**c**) selectivity characteristics of the PANI/TiO_2_ core−shell sensor (adapted from [93] with permission from the Elsevier B.V.); (**d**) schematic diagram of the rGO/TiO_2_/Au sensor; (**e**) response of the rGO/TiO_2_/Au sensor with UV−on and UV−off; (**f**) gas selectivity characteristics of the rGO/TiO_2_/Au sensor (adapted from [94] with permission from the American Chemical Society); (**g**) selectivity of the pure 2DPI, pure In_2_O_3,_ and the 2DPI/In_2_O_3_ gas sensor under various gases; (**h**) response of the pure 2DPI, pure In_2_O_3,_ and the 2DPI/In_2_O_3_ gas sensor with UV light irradiation; (**i**) long−term stability of the 2DPI/In_2_O_3_ gas sensor (adapted from [95] with permission from the Elsevier B.V.).

MOS and organic semiconductors were used to fabricate the ammonia (NH_3_) gas sensor. Safe et al. produced an NH_3_ gas sensor using a heterojunction of a p−type semiconductor polyaniline (PANI) and n−type semiconductor one−dimensional TiO_2_ nanofibers [93]. PANI, with a porous morphology, detected NH_3_ molecules selectively at room temperature, and TiO_2_ has a photocatalytic function. The PANI/TiO_2_ core−shell sensor, in which the two semiconductor materials were applied simultaneously, exhibited improved photocatalytic properties and selective NH_3_ gas response in a UV light irradiation. The PANI/TiO_2_ core−shell sensor was irradiated with UV at a wavelength of 365 nm to investigate the change in resistance. Figure 5a shows the resistance change in the sensor with a TiO_2_ core created from a material containing a 30% anatase− 70% rutile mixed crystal phase. The sensor shows improved photocatalytic characteristics compared to the sole rutile crystal phase. The PANI/TiO_2_ core−shell sensor was exposed to 50 ppb to 40 ppm NH_3_ gas, and the electrical response was measured. The PANI/TiO_2_ core−shell sensor had a response time of 63 s and a recovery time of 37 s when 1 ppm of NH_3_ gas was injected. Figure 5b shows the stability of the PANI/TiO_2_ core−shell sensor measured at 15−day intervals. The response of 1 ppm NH_3_ gas was measured under UV light irradiation. The gas sensor in which the core of TiO_2_ has an anatase−rutile phase showed an approximately 22% decreased response when exposed to UV light because of the reduced activity of the photocatalyst. In addition, the selective gas response performance for NH_3_ gas increased when the PANI/TiO_2_ core−shell sensor was irradiated with UV light (Figure 5c). UV irradiation improved the response time and recovery time of the PANI/TiO_2_ core−shell sensor. 

In 2018, Zhou et al. first reported a UV−enhanced NH_3_ detection sensor using graphene oxide nanosheets (rGO), TiO_2_ NPs, and Au NPs [94]. The rGO acted as the template to attach the TiO_2_ NPs and Au NPs and produced a high electron collector and transporter. The rGO with high conductivity detected changes in resistance effectively without heating. TiO_2_ NPs are photocatalytic materials that interact with UV light and NH_3_ molecules. Au NPs increase the sorption sites on the surface to detect sorption molecules and promote charge separation of electron–hole pairs generated by UV light. Figure 5d shows a schematic diagram of the rGO/TiO_2_/Au sensor. To fabricate the rGO/TiO_2_/Au sensor, Si/SiO_2_ was used for the substrate. Subsequently, Au (120 nm)/Ti (40 nm) electrodes were formed into planar interdigital shapes, and a conventional photolithography and lift−off process was used for patterning. In addition, the width of the electrode was 50 μm. The synthesized rGO/TiO_2_/Au solution was spray−coated on the prepared substrate and thermally treated in a vacuum oven. Figure 5e shows the NH_3_ gas response characteristics of the rGO/TiO_2_/Au sensor under UV irradiation (λ = 365 nm) and dark conditions. The response to NH_3_ gas is higher under UV irradiation than in the dark. The NH_3_ gas response of the rGO/TiO_2_/Au sensor with UV irradiation increased to 2.1% compared to the dark conditions. Additionally, response speed and recovery level were enhanced by 234 s and 43%, respectively. In addition, the selective characteristics of the rGO/TiO_2_/Au sensor were investigated. Figure 5f shows the gas response to NH_3_, H_2_S, CO, HCHO, and H_2_ gases. The selectivity for NH_3_ gas was approximately two times higher than that of the other gases. UV light irradiation enhanced the NH_3_ gas detection performance of the rGO/TiO2/Au sensor. In the dark, electrons from the rGO/TiO_2_/Au sensor flow from TiO_2_ NPs to Au NPs, producing a large−area depletion layer in rGO/TiO_2_ before being exposed to NH_3_ molecules. UV light irradiation induces more electrons to flow into Au NPs, resulting in a thicker depletion region. In addition, exposure of NH_3_ molecules to the rGO/TiO_2_/Au sensor interacts with more free electrons generated by UV light, resulting in an enhanced response.

The response of the gas sensor was improved by synthesizing organic materials and MOS. Yang et al. demonstrated an NH_3_ gas sensor using a composite material of two−dimensional polyimide (2DPI) and indium oxide (In_2_O_3_) [95]. Under UV light irradiation, the response of the 2DPI/In_2_O_3_ gas sensor was enhanced three times compared to the response of the gas sensor using only 2DPI or In_2_O_3_. 2DPI with photocatalytic characteristics promotes electron–hole separation and improves the response of In_2_O_3_−based gas sensors under UV illumination. The In_2_O_3_ with three−dimensional layered increases the adsorption sites through self−assembly properties. The NH_3_ gas response of the 2DPI/In_2_O_3_ gas sensor and pure 2DPI and In_2_O_3_ were compared. The response of the 2DPI/In_2_O_3_ gas sensor exposed to 10 ppm NH_3_ gas was approximately 6.9, which was higher than that of pure 2DPI and In_2_O_3_ gas sensors. The enhanced response of NH_3_ was due to the generated heterojunction between 2DPI and In_2_O_3_. Figure 5g shows the response of the 2DPI/In_2_O_3_ gas sensor to various gases. The 2DPI/In_2_O_3_ gas sensor has a higher NH_3_ gas selectivity characteristic than the two reference sensors mentioned above. Figure 5h shows the response of the pure 2DPI and In_2_O_3_ gas sensors and the 2DPI/In_2_O_3_ gas sensor under UV (λ = 365 nm) irradiation conditions. The 2DPI/In_2_O_3_ gas sensor was exposed to 1 ppm NH_3_ gas. The responses of the pure 2DPI and pure In_2_O_3_ gas sensors were 1.6 and 2.1, respectively. On the other hand, the response of the 2DPI/In_2_O_3_ gas sensor under UV light irradiation was 6.5, which was 3.66 times higher than the response in the dark. Figure 5i shows the long−term stability of the 2DPI/In_2_O_3_ gas sensor for ammonia gas detection. Over 150 days, 0.1, 0.5, 1, and 5 ppm of NH_3_ gas could be detected without degradation. The heterojunction of the 2DPI/In_2_O_3_ gas sensor improved the electrical conductivity. In Fermi level equilibrium, the electrons of 2DPI move into In_2_O_3_. In addition, oxygen ions are formed through oxygen in the air and free electrons. Subsequently, NH_3_ molecules with injected oxygen ions interact to reduce the sensor resistance. The energy of UV light is 3.4 eV, which is higher than the bandgap of the 2DPI/In_2_O_3_ composites (2.95 eV). Thus, photo−generated electrons from UV light cause more oxygen ion production, making the depletion layer smaller.

## 3. Visible−Activated Gas Sensors

### 3.1. Visible−Activated Nitric Oxide Gas Sensors

**Figure 6:**
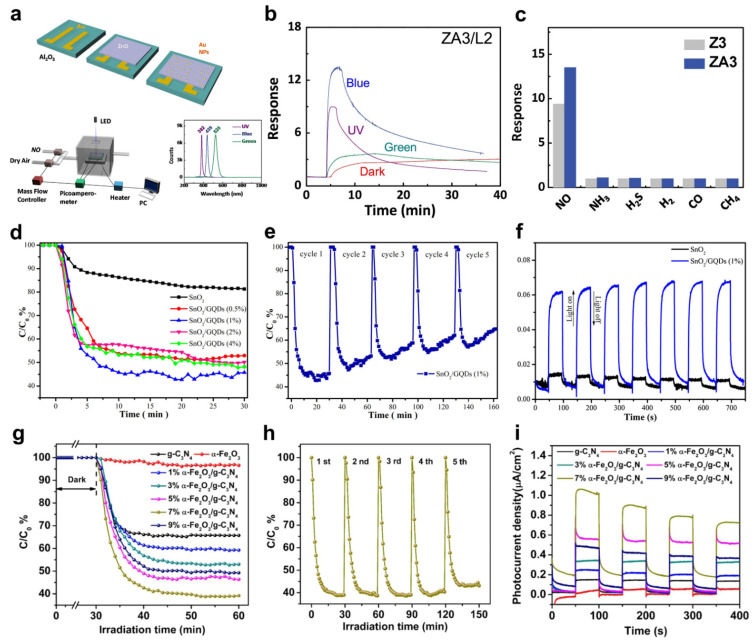
(**a**) Fabrication process of the Au/ZnO gas sensor; (**b**) response of the Au/ZnO gas sensor with various light irradiation (UV, blue, green); (**c**) selectivity characteristics of the Au/ZnO gas sensor (adapted from [96] with permission from the Elsevier B.V.); (**d**) photocatalytic spectrum of SnO_2_/GQDs under visible light irradiation; (**e**) recycling test for of SnO_2_/GQDs with visible light irradiation; (**f**) photocurrent density of the pure SnO_2_ and SnO_2_/GQDs (adapted from [97] with permission from the Elsevier B.V.); (**g**) photocatalytic performances of α−Fe_2_O_3_/g−C_3_N_4_ sensors with different composite ratios in removing NO gas; (**h**) recycling test for 7% SnO_2_/GQDs sensor with visible light irradiation; (**i**) photocurrent response of pure α−Fe_2_O_3_, g−C_3_N_4_, and α−Fe_2_O_3_/g−C_3_N_4_ (adapted from [98] with permission from the Elsevier B.V.).

In 2019, Chinh et al. reported a ZnO−based NO gas sensor loaded with an Au NP catalyst [96]. The energy of visible light irradiated to Au NPs reduced the barrier energy of ZnO, activating the adsorption and desorption of NO and oxygen molecules. A 2.5 mm × 2.5 mm Al_2_O_3_ substrate was prepared to fabricate the Au/ZnO gas sensor (Figure 6a). First, finger−shaped Au electrodes were deposited on the substrate. Diethyl zinc [Zn(C_2_H_5_)_2_] and H_2_O were then flowed alternately through ALD to deposit a ZnO thin film. Subsequently, an annealing treatment was performed at 500 °C for two hours. The Au NPs were loaded on the ZnO thin film by immersing the films in an Au colloidal solution and annealing them at 500 °C. The wavelengths of the light−emitting diode (LED) used to measure the Au/ZnO gas sensor were ultraviolet (λ = 382 nm), blue (λ = 439 nm), and green (λ = 525 nm), and light with an intensity of 0.76 mW/cm^2^ was irradiated. Figure 6b shows the response of the Au/ZnO gas sensor to various wavelengths of light when exposed to 10 ppm NO gas. The Au/ZnO gas sensor exhibited the strongest response of more than 12 in blue light irradiation. In addition, the resistance of the Au/ZnO gas sensor was increased because the Schottky contact was bent more. Figure 6c shows the selectivity of the Au/ZnO gas sensor. The response of 150 ppm of NH_3_, H_2_S, H_2_, CO, and CH_4_ gases was investigated. On the other hand, the responses to other gases were lower than that of 10 ppm NO gas.

To fabricate NO gas sensors with improved reactivity under visible light irradiation, Xie et al. used tin oxide (SnO_2_) and graphene quantum dots (GQDs) [97]. SnO_2_, an n−type MOS, has a bandgap of 3.60 eV and is used as a core material in gas sensors because of its excellent electrical and optical characteristics. Nevertheless, it is difficult to use visible light for a SnO_2_−based sensor with a wide bandgap. Hence, SnO_2_ and GQDs with a narrow bandgap material were combined to fabricate high−response gas sensors. The GQDs improved the charge separation efficiency when irradiated with visible light. The photocatalytic characteristics were investigated according to the GQD content. Figure 6d shows the photocatalytic spectrum for the NO gas under visible light irradiation. All samples were exposed to 600 ppb of NO gas, and the adsorption−desorption state was equilibrated by exposure to the NO gas for 30 minutes before visible light irradiation. The NO removal rate with only SnO_2_ was 18%, whereas it increased dramatically when GQDs were deposited on SnO_2_. The rate of NO gas removal reached 57% when the amount of GQDs was 1%. On the other hand, excessive deposition of GQDs reduces light efficiency because it blocks the active site of SnO_2_. Figure 6e shows a cycle test for the NO gas under visible light irradiation. During the five cycles, the degradation product accumulated and deteriorated the photoactivity. Figure 6f shows the photocurrent densities of the pure SnO_2_ and SnO_2_/GQDs under visible light irradiation. The improved charge separation efficiency due to QGD increased the response of the SnO_2_/GQDs sensor under visible light illumination.

In 2021, Geng et al. reported a heterojunction−structured NO gas sensor using carbon nitride (g−C_3_N_4_) and hematite (α−Fe_2_O_3_) [98]. g−C_3_N_4_ is a photocatalytic material but shows poor performance for NO removal gas due to rapid electron−hole recombination and low light harvesting ability. The photocatalytic efficiency was increased by combining α−Fe_2_O_3_ with g−C_3_N_4_ to achieve a heterojunction structure of the Z−scheme. The presented 2D/2D structure has a high interfacial area and active sites for interacting with NO molecules and effectively absorbing visible light to promote charge separation and transport. Figure 6g shows the photocatalytic performance of the α−Fe_2_O_3_/g−C_3_N_4_ sensors with different composite ratios of NO removal. The α−Fe_2_O_3_/g−C_3_N_4_ sensor was irradiated with visible light using a Xe lamp with a 400 nm cutoff filter applied. The NO gas removal performance of pure α−Fe_2_O_3_ and g−C_3_N_4_ was 3.4% and 34.2%, respectively. On the other hand, the NO gas degradation efficiency increased when the heterojunction was formed. Overall, the charge separation efficiency increased with increasing α−Fe_2_O_3_ content. The highest NO gas degradation efficiency of 60.8% was observed when the α−Fe_2_O_3_ content was 7%. Figure 6h shows the NO gas removal rate of 7% α−Fe_2_O_3_/g−C_3_N_4_ over five cycles. The optimized α−Fe_2_O_3_ content barely changed the NO removal efficiency and stabilized the sensor. Figure 6i shows the photocurrent response of various samples. Similarly, pure α−Fe_2_O_3_ and g−C_3_N_4_ had the lowest photocurrent density. However, 7% α−Fe_2_O_3_/g−C_3_N_4_ had the highest photocurrent density. An appropriate α−Fe_2_O_3_ content produces a g−C_3_N_4_ active area with enhanced NO gas degradation efficiency and photocurrent density.

### 3.2. Visible−Activated Nitric Dioxide Gas Sensors

**Figure 7:**
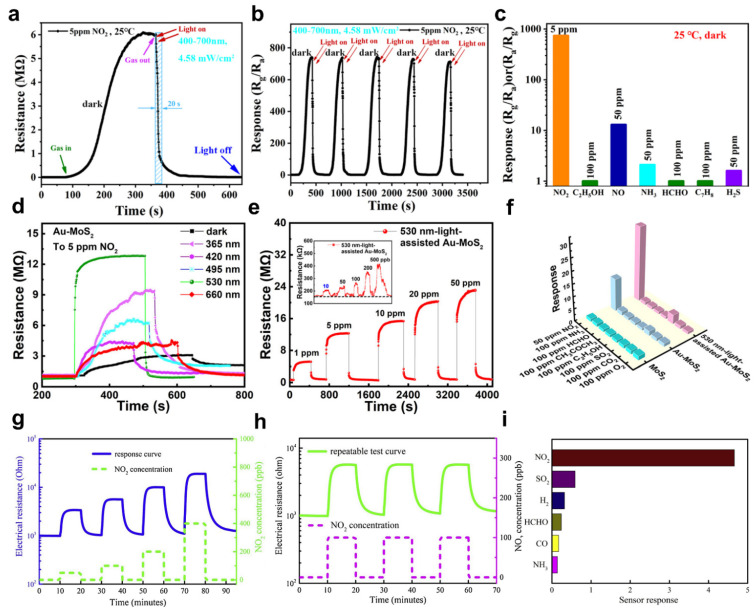
(**a**) Response of the In_2_O_3_ NWs gas sensor with visible light irradiation; (**b**) stability characteristics of the In_2_O_3_ NWs gas sensor; (**c**) selectivity characteristics of the In_2_O_3_ NWs gas sensor (adapted from [99] with permission from the Elsevier B.V.); (**d**) response of the Au−MoS_2_ gas sensor with UV and visible light; (**e**) response of the Au−MoS_2_ gas sensor with various NO_2_ gas concentration; (**f**) selectivity characteristics of the Au−MoS_2_ gas sensor (adapted from [100] with permission from the American Chemical Society); (**g**) electrical resistance response of the rGO@ZnO sensor with white light illumination; (**h**) repeated NO_2_ gas response of the rGO@ZnO sensor; (**i**) selectivity characteristics of the rGO@ZnO sensor (adapted from [101] with permission from the Elsevier B.V.).

In_2_O_3_ nanowires (NWs) synthesized by electrospinning improve the responsivity of gas sensors with a high surface area. Zhang et al. produced a NO_2_ gas sensor based on In_2_O_3_ nanowires (NWs) [99]. In_2_O_3_ is an n−type MOS with a bandgap of 2.8 eV and high conductivity that exhibits responsivity and selectivity to NO_2_ gas. The In_2_O_3_ NWs have a high surface area and a loose arrangement, which promotes the interaction between the NO_2_ molecules and oxygen species. Defects in the In_2_O_3_ NWs and oxygen species absorbed on the surface increase the responsivity to NO_2_ gas. On the other hand, the In_2_O_3_ NWs gas sensor has a long recovery time in the dark. To achieve a rapid recovery time, irradiating visible light promotes the removal of adsorbed NO_2_ gas from the In_2_O_3_ NWs gas sensor. Figure 7a shows the response of the In_2_O_3_ NWs gas sensor when exposed to 5 ppm of NO_2_ gas. Visible light has a wavelength range of 400 to 700 nm and was irradiated with an intensity of 4.58 mW/cm^2^. The resistance was restored to 90% of the initial value in approximately 20 s when the In_2_O_3_ NWs gas sensor was irradiated by visible light, and at the same time, the NO_2_ gas flow was stopped. In addition, repeated tests were performed to confirm the stability of the In_2_O_3_ NWs gas sensor (Figure 7b). The In_2_O_3_ NWs gas sensor was controlled in visible light, and the changed resistance reached the initial value during five cycles without deterioration. Figure 7c shows the selectivity characteristics of the In_2_O_3_ NWs gas sensor. The response to 5 ppm NO_2_ gas was highest at 740. On the other hand, the responses to ethanol, formaldehyde, and toluene gas were negligible. In addition, NO, NH_3,_ and H_2_S gases showed stronger responses than volatile organic compounds (VOCs) but were significantly lower than NO_2_ gas. The initial resistance of the In_2_O_3_ NWs gas sensor is determined by the oxygen species adsorbed on the surface. When the In_2_O_3_ NWs gas detectors were exposed to NO_2_ gas, oxygen−related gases on the surface and electrons extracted from the conduction band of In_2_O_3_ NWs interacted and increased the resistance. On the other hand, photons generated by visible light irradiation desorb the oxygen ions from the surface. Thus, the released electrons were transported to In_2_O_3_ nanowires, reducing the resistance.

To enhance the detection limit of a MoS_2_−based NO_2_ gas sensor, Chen et al. loaded Au NPs on MoS_2_ to use the localized surface plasmon resonance (LSPR) effect [100]. The decorated Au NPs irradiated with optimized light, the strong absorption of light, and the enhanced electromagnetic near−field due to the LSPR effect increase the light absorption efficiency of MoS_2_. In addition, Au−MoS_2_, with its high surface−to−volume ratio structure, provides more opportunity to interact with NO_2_ gas. Figure 7d shows the response of the Au−MoS_2_ gas sensor to the NO_2_ gas with various wavelengths of light (λ = 365, 420, 495, 530, and 660 nm) irradiated. The power of the visible light source irradiated to the Au−MoS_2_ gas sensor was the same as 10 W, and 5 ppm NO_2_ gas was injected. Visible light at 530 nm was optimized for NO_2_ gas detection. The frequency of visible light at 530 nm and the vibration frequency of Au NPs correspond. Au NPs absorb more photon energy because of the matched frequency. As a result, the Au−MoS_2_ gas sensor has an LSPR effect with strong absorption for 530 nm visible light. In addition, the response to different concentrations of NO_2_ gas was investigated (Figure 7e). The Au−MoS_2_ gas sensor detected 10 ppb to 50 ppm NO_2_ gas, and the irradiated light improved the detection limit of NO_2_ gas. Figure 7f shows the selectivity of the pure MoS_2_ and Au−MoS_2_ gas sensors in the dark and under visible light illumination. The pure MoS_2_−based gas sensor showed the weakest response to all gases. On the other hand, the response to NO_2_ gas increased when loaded with Au NPs. In addition, irradiation with 530 nm visible light further enhanced the gas response to NO_2_ gas.

In 2021, Geng et al. synthesized reduced graphene (rGO) and the used oxygen−deficient zinc oxide (ZnO_1−x_) composites using hydrothermal methods. Based on these combinations, they produced an NO_2_ gas detector operating at ambient temperature [101]. Donor defects generated during synthesis narrow the bandgap of ZnO, allowing it to respond to visible light. In addition, the rGO effectively absorbs light with its large surface area and electron mobility. The p–n junction produced at the ZnO interface attached to the rGO enhanced the NO_2_ sensing response to white light. Figure 7g shows the electrical resistance response of the rGO@ZnO sensor to 50 to 400 ppb of NO_2_ gas. The rGO@ZnO sensor was irradiated with white light with an intensity of 0.15 W/cm^2^. The rGO@ZnO sensor showed improved responsivity compared to the pure ZnO sensor. The pure ZnO sensor had a response of 0.19 at 50 ppb NO_2_ gas, while the rGO@ZnO sensor showed a response of 2.31. The increased response to low−concentration NO_2_ gas improved the detection limit of the rGO@ZnO sensor. Figure 7h shows the response to the 100 ppb NO_2_ gas repeated test. The rGO@ZnO sensor under white light irradiation showed repeated sensing without significant degradation. In addition, the selectivity characteristics of the rGO@ZnO sensor were investigated (Figure 7i). The concentrations of SO_2_, CO, and NH gases injected into the rGO@ZnO sensor were 100 ppm. In addition, 400 ppm H_2_ gas and 10 ppm HCHO gas were injected. Finally, the concentration of NO_2_ gas was 100 ppb, which was the lowest concentration but led to the strongest response.

### 3.3. Visible−Activated Formaldehyde Gas Sensors

**Figure 8:**
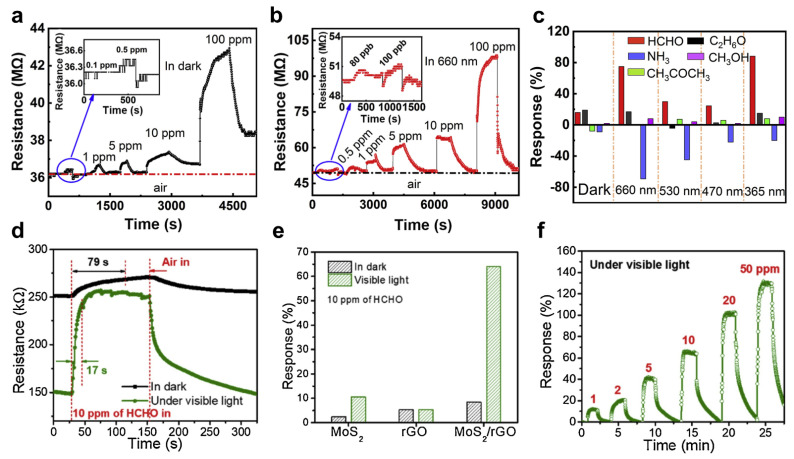
(**a**) Response of the HoFeO_3_ gas sensor under dark and various visible light; (**b**) response of the HoFeO_3_ gas sensor under red light irradiation; (**c**) selectivity characteristics of the HoFeO_3_ gas sensor (adapted from [102] with permission from the Elsevier B.V.); (**d**) response of the MoS_2_/rGO hybrid gas sensor in the dark condition and visible light irradiation; (**e**) responses of pure MoS_2_, rGO, and MoS_2_/rGO hybrid gas sensors; (**f**) response of the MoS_2_/rGO hybrid gas sensor to 1–50 ppm HCHO gas (adapted from [103] with permission from the Elsevier B.V.).

In 2021, Song et al. produced an HCHO gas detector using P−type material, HoFeO_3_ NPs, with high responsivity in light at various wavelengths (λ = 365, 470, 530, and 660 nm) [102]. In particular, the HoFeO_3_ gas sensor showed the most enhanced response to red light (λ = 660 nm). Figure 8a shows the HCHO gas response of the HoFeO_3_ gas sensor in the dark under and various light illuminations. The HoFeO_3_ gas detectors were exposed to 100 ppb to 100 ppm HCHO gas. The lowest detection limit of the HoFeO_3_ gas sensor was 0.5 ppm, and the response was 0.64. On the other hand, the response to HCHO gas was improved when the HoFeO_3_ gas sensor was irradiated with red light. Figure 8b shows the response of the HoFeO_3_ gas sensor to red light irradiation. Compared to the dark condition, the response to HCHO gas improved under red light irradiation, and the response was 1.9 when exposed to 80 ppb HCHO gas. The selectivity of the HoFeO_3_ gas sensor was investigated (Figure 8c). The HCHO, C_2_H_6_O, CH_3_COCH_3_, NH_3_, and CH_3_OH gases with concentrations of 100 ppm were injected into the HoFeO_3_ gas sensor. In particular, the HCHO gas showed a high response when irradiated with 660 nm and 365 nm light. In addition, the HoFeO_3_ gas sensor had a strong response to NH_3_. The HoFeO_3_ gas sensor showed decreased resistance with NH_3_ gas but increased resistance when exposed to HCHO gas. Thus, the two gases could be distinguished owing to their opposite tendency to change the resistance.

The synergy of TMDs and graphene improved HCHO detection performance under visible light irradiation. In 2020, Wang et al. reported a MoS_2_/rGO−based HCHO gas sensor [103]. MoS_2_ acts as a photocatalyst material and oxidizes HCHO gas to CO_2_ and H_2_O when irradiated with visible light. In addition, rGO induces efficient charge separation. Figure 8d shows the response of the MoS_2_/rGO hybrid gas sensor irradiated with visible light (λ > 420 nm) to 10 ppm HCHO gas. In addition, the responsivity under dark conditions was measured to compare to visible light irradiation conditions. When irradiated with visible light, the response time of the MoS_2_/rGO hybrid gas sensor was 17 s, which is approximately 62 s less than in the dark condition. In addition, the resistance to HCHO gas increased. The initial resistance of the MoS_2_2/rGO hybrid gas sensor under visible light irradiation was 149 kΩ, which increased by 262 kΩ upon exposure to HCHO gas, resulting in an improved response of 64%. On the other hand, the response to HCHO gas was improved by 8.5% under dark conditions. The sensing responses of pure MoS_2_, rGO, and MoS_2_/rGO hybrid gas sensors were compared (Figure 8e). The response under dark and visible light conditions was compared with 10 ppm of injected HCHO gas. Although pure MoS_2_ and rGO gas sensors have less than approximately 12% response, the MoS_2_/rGO hybrid gas sensor with both materials applied has more than 60% response in visible light. Figure 8f shows the response of the MoS_2_/rGO hybrid gas sensor to exposure to 1 to 50 ppm HCHO gas. The MoS_2_/rGO hybrid gas sensor exhibited a 12.6% response to 1 ppm of HCHO gas and 126.8% at 50 ppm.

### 3.4. Visible−Activated Ammonia Gas Sensors

**Figure 9:**
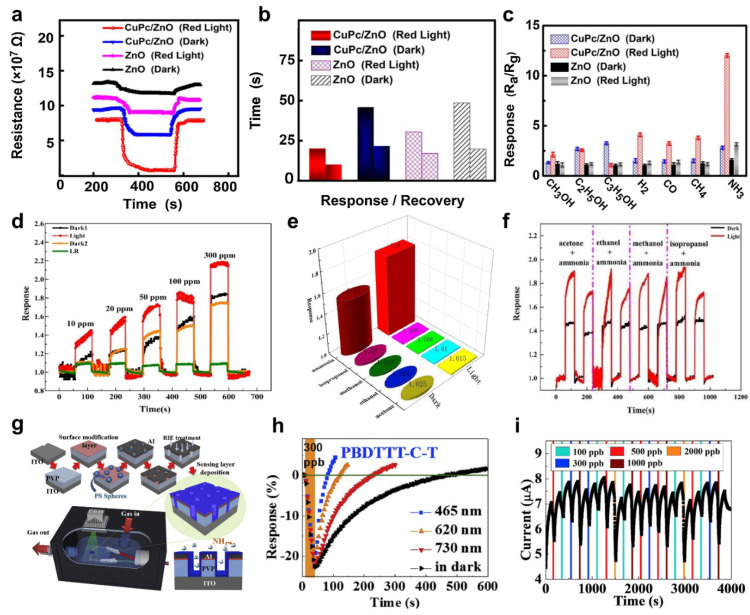
(**a**) Response of the CuPc/ZnO gas sensor and the pure ZnO gas sensor in dark and under red light irradiation; (**b**) response and recovery time of the CuPc/ZnO gas sensor and the pure ZnO gas sensor under dark and red light irradiation; (**c**) selectivity characteristics of the CuPc/ZnO gas sensor (adapted from [104] with permission from the Elsevier B.V.); (**d**) response of the Ag_3_PO_4_ NPs gas sensor; (**e**) selectivity characteristics of the Ag_3_PO_4_ NPs gas sensor; (**f**) response of the mixed NH_3_–based gas (adapted from [105] with permission from the John Wiley and Sons); (**g**) fabrication process of the PBDTTT−C−T gas sensor and the NH_3_ gas detection system; (**h**) response of the PBDTTT−C−T gas sensor; (**i**) stability of the PBDTTT−C−T gas sensor (adapted from [106] with permission from the Elsevier B.V.).

In 2021, Huang et al. demonstrated a copper phthalocyanine−loaded zinc oxide (CuPc/ZnO) −based NH_3_ gas sensor fabricated using a microwave−assisted hydrothermal synthesis method [104]. The responsivity of the CuPc/ZnO gas sensor with NH_3_ gas was improved under red light irradiation (λ = 600 nm to 622 nm). The red−light irradiation effect promoted the desorption of oxygen on the ZnO surface, making the depletion layer thin. In addition, the photoelectrons generated by red light increased the electron concentration in ZnO, resulting in more oxygen ions that interact with the NH_3_ molecules. Figure 9a shows the NH_3_ gas response of the CuPc/ZnO gas sensor and the pure ZnO gas sensor. The concentration of injected NH_3_ gas was 80 ppm, and the red light had an intensity of 0.15 W/cm^2^. The pure ZnO gas sensor and the CuPc/ZnO gas sensor showed a further decrease in resistance when irradiated with red light than in the dark. On the other hand, the CuPc/ZnO gas sensor showed improved response and recovery times, as well as a response to NH_3_ gas compared to the pure ZnO gas sensor (Figure 9b). The red−light illumination effect reduced the barrier height of the CuPc/ZnO gas sensor to enhance the electron mobility. The response time and recovery time values of the pure ZnO were measured at 31 s and 18 s, respectively. On the other hand, the CuPc/ZnO gas sensor exhibited a response time value of 20 s and a recovery time value of 10 s. Figure 9c shows the response behavior of the CuPc/ZnO gas sensor and the pure ZnO gas sensor to various gases. The selectivity of CH_3_OH, C_2_H_5_OH, C_3_H_5_OH, H_2_, CO, and CH_4_ at 80 ppm was investigated. The response of the CuPc/ZnO gas sensor irradiated with red light to NH_3_ gas was approximately 12, whereas the responses to the remaining gases were less than five. 

For achieving high responsivity of NH_3_ gas sensor, Shao et al. used silver phosphate (Ag_3_PO_4_) as a photocatalytic [105]. The Ag_3_PO_4_ NPs have efficient separation of electron–hole pairs with visible light illumination. The calculated bandgap and intrinsic absorption long wavelength limit of Ag_3_PO_4_ NPs synthesized by the precipitation method was 2.24 eV and 554 nm, respectively, which is less energy than the white LED with a wavelength range of 440 nm to 470 nm. Figure 9d shows the response of the Ag_3_PO_4_ NPs gas sensor to NH_3_ gas with concentrations ranging from 10 to 300 ppm. Dark1 and Light1 indicate the responses of the Ag_3_PO_4_ NPs gas sensor to NH_3_ gas with and without visible light illumination, respectively. Owing to the visible light activated Ag_3_PO_4_ NPs, the resistance change in the Ag_3_PO_4_ NPs gas sensor was larger under visible light illumination. In addition, when the Ag_3_PO_4_ NPs were exposed to light, they could corrode and generate metal silver on the surface, affecting the gas sensor’s performance. Dark 2 is the response of the Ag_3_PO_4_ NPs gas sensor in the dark, which was previously exposed to visible light to NH_3_ gas. The response of Dark 2 was similar that in Dark 1 because of the suppressed photocorrosion of Ag_3_PO_4_ by adjusting the visible light exposure time. The LR is the calculated Ag_3_PO_4_ NPs gas sensor response under visible light without NH_3_ gas. Figure 9e shows the selectivity characteristics of the Ag_3_PO_4_ NPs gas sensor. The Ag_3_PO_4_ NPs gas sensor was exposed to IPA, methanol, ethanol, gases, and NH_3_ at 100 ppm each. The response of the Ag_3_PO_4_ NPs gas sensor to NH_3_ gas was highly selective, 1.55 and 1.8 in the dark and visible light conditions, respectively. In addition, the response of mixed NH_3_–based gases were investigated (Figure 9f). The Ag_3_PO_4_ NPs gas sensor has strong selectivity for NH_3_ molecules as a result of its response to NH_3_based mixed gases.

Visible light was irradiated to shorten the recovery time of organic material−based gas sensors. Lin et al. reported a NH_3_ gas detector with a vertical diode by using poly [[4,8−bis[5−(2−ethylhexyl)−2−thienyl]benzo[1,2−b:4,5−b’]dithiophene−2,6−diyl][2−(2ethy l−1oxohexyl)thieno[3,4−b]thiophenediyl]] (PBDTTT−C−T) [106]. The PBDTTT−C−T gas detector based on the organic materials was irradiated with various wavelengths of visible light to compensate for the slow recovery time. On the other hand, the recovery time was improved when the PBDTTT−C−T gas sensor was irradiated with blue light because of the current compensation effect. Figure 9g shows the fabrication process of the PBDTTT−C−T gas sensor and the NH_3_ gas detection system. ITO with improved surface hydrophilic characteristics through the oxygen plasma treatment was used as a substrate. Subsequently, poly(4−vinylphenol) (PVP) for the insulating layer was deposited by spin coating. Poly(3−hexylthiophene−2,5−diyl) (P3HT) was spin−coated for the adhesion of polystyrene (PS) nano−spheres. The prepared samples were immersed in a PS solution to form polystyrene (PS) nano−spheres. Al was deposited over the entire surface using the evaporation method, and the PS spheres were removed using 3M Scotch tape to induce porous patterning on the sample. The exposed PVP was removed by oxygen plasma etching. Finally, PBDTTT−C−T was deposited using the blade coating method. The PBDTTT−C−T gas sensor was mounted in an opaque box, and the wavelengths of the LED used were 465, 620, and 730 nm. Figure 9h shows the response of the PBDTTT−C−T gas sensor to 300 ppb of NH_3_ gas. After NH_3_ gas was injected into the PBDTTT−C−T gas sensor for 30 s, it was irradiated with the LED. As a result, the recovery time was improved when the PBDTTT−C−T gas sensor was irradiated with LED light. In addition, the enhanced response to light at 465 nm was attributed to the stronger irradiance than the other two LEDs. Figure 9i shows the response of the PBDTTT−C−T gas sensor to NH_3_ at concentrations from 100 to 2000 ppb. The stability of the PBDTTT−C−T gas sensor was confirmed by performing at least three tests with different NH_3_ gas concentrations. Table 1 summarizes the sensing performance of photo−activated gas sensors for NO, NO_2_, HCHO, and NH_3_ based on UV and visible light.

## 4. Conclusions

This review reported various strategies for producing gas detectors activated by ultraviolet and visible light that have been reported in recent years. Research on photo−activated gas detectors using metal oxide semiconductors, TMDs, and carbon nanotubes has been reported. Techniques, such as porous structures, heterojunctions, and surface defects, improve the responsivity, selectivity, response, and recovery time of photo−activated gas detectors. In addition, photo−activated gas detectors are classified according to the type of detectable gas, such as nitric oxide, nitric dioxide, formaldehyde, and ammonia gas. Photo−activated gas detectors, with external light illumination, replaced conventional high−temperature gas detectors, providing a technological foundation for low−power consumption and miniaturization. On the other hand, despite these advantages, photo−activated gas sensing technology still has technical barriers to overcome, and technology development strategies for advancing high−performance light−activated gas detectors are needed.

1. Photo−activated gas detectors use a specific wavelength of light to increase selectivity for a target gas. However, most of the photoactive gas detector performance has been reported in an environment where the variables are controlled, such as temperature and humidity. Therefore, to commercialize photoactive gas detectors in the industry, measurements should be performed in an environment with complex variables, including detecting a target gas contained in a mixed gas.
2. The photo−activated gas detection system should reduce its weight and size for portability and miniaturization. The presence of light sources that irradiate ultraviolet or visible light has expanded the use of photo−activated gas detection systems. In addition, the integration of existing systems, such as computers and smartphones, should be considered. Therefore, it is necessary to re−examine the photo−activated gas detection system using a new structural design.
3. The sustainability and stability of photoactive detectors need to be reviewed further. Gas detectors are attracting attention as a safety system in various fields, such as factories and hospitals. To monitor a gas in real time, it is necessary to ensure the overall durability and reliability of the photoactive gas detectors.
4. The presence or absence of a disease could be diagnosed through the concentration of a target gas detected in human exhalation. On the other hand, it is necessary to detect low concentrations of gases precisely to distinguish between healthy and diseased people. The photoactive gas detectors for diagnosing disease require an improved response to low−concentration gases.

In spite of the aforementioned technical limitations, research and technological development of photo−activated gas detectors are actively progressing. Photo−activated gas detectors have potential advantages for gas detection and are believed to be core electronics for industrial stabilization in the future.

## Figures and Tables

**Table 1:** Comparison of sensing performance of the photo−activated gas sensor.

Materials	Light	Target Gas	Limit of Detection	Sensitivity	Ref
TiO_2_@NGQDs	UV (λ = 365 nm)	Nitric oxide	10 ppm	~31.1% at 100 ppm	[85]
Cu−TCA/TiNCs	UV (λ = 365 nm)	Nitric oxide	140 ppb	~124% at 50 ppm	[86]
ZNO NW	UV (λ = 325 nm)	Nitric dioxide	20 ppb	~708% at 1 ppm	[87]
MoS_2_	UV (λ = 365 nm)	Nitric dioxide	5 ppm	~3% at 100 ppm	[88]
CNT	UV (λ = 365, 275 nm)	Nitric dioxide	1 ppm	N/A	[89]
Au−ZnO POH	UV (λ = 365nm)	Formaldehyde	50 ppm	~7.6 at 100 ppm	[90]
SnO_2_@TiO_2_	UV (λ = 365nm)	Formaldehyde	100 ppb	~32.5% at 10 ppm	[91]
NiS/Ni−ZnO	UV (λ = 365nm)	Formaldehyde	2 ppm	~330% at 10 ppm	[92]
PANI/TiO_2_	UV (λ = 365 nm)	Ammonia	50 ppb	~109.87% at 1 ppm	[93]
rGO/TiO_2_/Au	UV (λ = 365 nm)	Ammonia	2 ppm	~8.9% at 50 ppm	[94]
2DPI/In_2_O_3_	UV (λ = 365 nm)	Ammonia	50 ppb	~6.5 at 1 ppm	[95]
Au/ZnO	Vis (λ = 382, 439, 525 nm)	Nitric oxide	1 ppm	~ 12 at 10 ppm, blue	[96]
SnO_2_/GQDs	Vis (λ ≥ 420 nm)	Nitric oxide	600 ppb	N/A	[97]
α−Fe_2_O_3_/ g−C_3_N_4_	Vis (λ ≥ 400 nm)	Nitric oxide	600 ppb	N/A	[98]
In_2_O_3_ NW	Vis (λ = 400 to 700 nm)	Nitric dioxide	10 ppb	~ 750% at 5 ppm	[99]
Au/MoS_2_	Vis (λ = 530 nm)	Nitric dioxide	10 ppb	~8.1 at 1 ppm	[100]
rGO@ZnO	Vis, white LED	Nitric dioxide	50 ppb	~2.31 at 50 ppb	[101]
HoFeO_3_	Vis (λ = 660 nm)	Formaldehyde	80 ppb	~78% at 100 ppm	[102]
MoS_2_/rGO	Vis (λ > 420 nm)	Formaldehyde	20 ppb	~64% at 10 ppm	[103]
CuPc/ZnO	Vis (λ = 600 to 622 nm)	Ammonia	800 ppb	~12 at 80 ppm	[104]
Ag_3_PO_4_ NP	Vis (λ = 400 to 800 nm)	Ammonia	10 ppm	~45% at 10 ppm	[105]
PBDTTT−C−T	Vis (λ = 465, 620, 730 nm)	Ammonia	100 ppb	~22 at 300 ppb, blue	[106]

## References

[1] Kaniyoor A., Jafri R.I., Arockiadoss T., Ramaprabhu S. (2009). Nanostructured Pt decorated graphene and multi walled carbon nanotube based room temperature hydrogen gas sensor. Nanoscale.

[2] Lupan O., Chai G., Chow L. (2008). Novel hydrogen gas sensor based on single ZnO nanorod. Microelectron. Eng..

[3] Wong Y.M., Kang W.P., Davidson J.L., Wisitsora-At A., Soh K.L. (2003). A novel microelectronic gas sensor utilizing carbon nanotubes for hydrogen gas detection. Sens. Actuators B Chem..

[4] Xing X., Chen T., Zhao R., Wang Z., Wang Y. (2018). A low temperature butane gas sensor used Pt nanoparticles-modified AZO macro/mesoporous nanosheets as sensing material. Sens. Actuators B Chem..

[5] Liu X., Pan K., Wang L., Dong C., Xiao X., Wang Y. (2015). Butane detection: W-doped TiO 2 nanoparticles for a butane gas sensor with high sensitivity and fast response/recovery. RSC Adv..

[6] Kim J.C., Jun H.K., Huh J.-S., Lee D.D. (1997). Tin oxide-based methane gas sensor promoted by alumina-supported Pd catalyst. Sens. Actuators B Chem..

[7] Waitz T., Wagner T., Sauerwald T., Kohl C., Tiemann M. (2009). Ordered mesoporous In_2_O_3_: Synthesis by structure replication and application as a methane gas sensor. Adv. Funct. Mater..

[8] Niu L., Luo Y., Li Z. (2007). A highly selective chemical gas sensor based on functionalization of multi-walled carbon nanotubes with poly(ethylene glycol). Sens. Actuators B Chem..

[9] Sholehah A., Karmala K., Huda N., Utari L., Septiani N.L.W., Yuliarto B. (2021). Structural effect of ZnO-Ag chemoresistive sensor on flexible substrate for ethylene gas detection. Sens. Actuators A Phys..

[10] Seddik B., Salima B., Houda G. (2021). Fe doped ZnO nanostructures prepared via sol-gel dip-coating technique for iso-butane (i-C4H10) sensing. Mater. Today Commun..

[11] Guillén-Bonilla H., Flores-Martínez M., Rodríguez-Betancourtt V.-M., Guillen-Bonilla A., Reyes-Gómez J., Gildo-Ortiz L., de la Luz Olvera Amador M., Santoyo-Salazar J. (2016). A novel gas sensor based on MgSb2O6 nanorods to indicate variations in carbon monoxide and propane concentrations. Sensors.

[12] Morán-Lázaro J.P., Guillen-López E.S., López-Urias F., Muñoz-Sandoval E., Blanco-Alonso O., Guillén-Bonilla H., Guillén-Bonilla A., Rodríguez-Betancourtt V.M., Sanchez-Tizapa M., Olvera-Amador M.d.l.L. (2018). Synthesis of ZnMn2O4 nanoparticles by a microwave-assisted colloidal method and their evaluation as a gas sensor of propane and carbon monoxide. Sensors.

[13] Latyshev V.M., Berestok T.O., Opanasyuk A.S., Kornyushchenko A.S., Perekrestov V.I. (2017). Nanostructured ZnO films for potential use in LPG gas sensors. Solid State Sci..

[14] Shinde V.R., Gujar T.P., Lokhande C.D., Mane R.S., Han S.-H. (2007). Use of chemically synthesized ZnO thin film as a liquefied petroleum gas sensor. Mater. Sci. Eng. B.

[15] Fu Y., Nie Y., Zhao Y., Wang P., Xing L., Zhang Y., Xue X. (2015). Detecting liquefied petroleum gas (LPG) at room temperature using ZnSnO3/ZnO nanowire piezo-nanogenerator as self-powered gas sensor. ACS Appl. Mater. Interfaces.

[16] Wang L., Wang S., Xu M., Hu X., Zhang H., Wang Y., Huang W. (2013). A Au-functionalized ZnO nanowire gas sensor for detection of benzene and toluene. Phys. Chem. Chem. Phys..

[17] Li Z., Huang Y., Zhang S., Chen W., Kuang Z., Ao D., Liu W., Fu Y. (2015). A fast response & recovery H2S gas sensor based on α-Fe2O3 nanoparticles with ppb level detection limit. J. Hazard. Mater..

[18] Vessally E., Behmagham F., Massoumi B., Hosseinian A., Edjlali L. (2016). Carbon nanocone as an electronic sensor for HCl gas: Quantum chemical analysis. Vacuum.

[19] Aguir K., Lemire C., Lollman D.B.B. (2002). Electrical properties of reactively sputtered WO3 thin films as ozone gas sensor. Sens. Actuators B Chem..

[20] Da Silva L.F., Catto A.C., Avansi W., Cavalcante L.S., Andrés J., Aguir K., Mastelaro V.R., Longo E. (2014). A novel ozone gas sensor based on one-dimensional (1D) α-Ag_2_WO_4_ nanostructures. Nanoscale.

[21] Zhou Q., Zeng W., Chen W., Xu L., Kumar R., Umar A. (2019). High sensitive and low-concentration sulfur dioxide (SO_2_) gas sensor application of heterostructure NiO-ZnO nanodisks. Sens. Actuators B Chem..

[22] Luo Y., Zhang D., Fan X. (2020). Hydrothermal Fabrication of Ag-Decorated MoSe₂/Reduced Graphene Oxide Ternary Hybrid for H₂S Gas Sensing. IEEE Sens. J..

[23] Leidinger M., Rieger M., Sauerwald T., Alépée C., Schütze A. (2016). Integrated pre-concentrator gas sensor microsystem for ppb level benzene detection. Sens. Actuators B Chem..

[24] Thota C., Modigunta J.K.R., Reddeppa M., Park Y.H., Kim H., Kang H., Kokkiligadda S., Lee S., Murali G., Park S.Y. (2022). Light stimulated room-temperature H2S gas sensing ability of Cl-doped carbon quantum dots supported Ag nanoparticles. Carbon N. Y..

[25] Reddeppa M., Park B.-G., Murali G., Choi S.H., Chinh N.D., Kim D., Yang W., Kim M.-D. (2020). NOx gas sensors based on layer-transferred n-MoS_2_/p-GaN heterojunction at room temperature: Study of UV light illuminations and humidity. Sens. Actuators B Chem..

[26] Mahalingam A., Naayagi R.T., Mastorakis N.E. (2012). Design and implementation of an economic gas leakage detector. Recent Res. Appl. Electr. Comput. Eng..

[27] Brunelli D., Rossi M. (2014). Enhancing lifetime of WSN for natural gas leakages detection. Microelectron. J..

[28] Aalsalem M.Y., Khan W.Z., Gharibi W., Khan M.K., Arshad Q. (2018). Wireless Sensor Networks in oil and gas industry: Recent advances, taxonomy, requirements, and open challenges. J. Netw. Comput. Appl..

[29] Suma V., Shekar R.R., Akshay K.A. (2019). Gas leakage detection based on IOT. Proceedings of the 2019 3rd International conference on Electronics, Communication and Aerospace Technology (ICECA).

[30] Rostrup-Nielsen J.R. (1993). Production of synthesis gas. Catal. today.

[31] Galvita V.V., Semin G.L., Belyaev V.D., Semikolenov V.A., Tsiakaras P., Sobyanin V.A. (2001). Synthesis gas production by steam reforming of ethanol. Appl. Catal. A Gen..

[32] York A.P.E., Xiao T., Green M.L.H., Claridge J.B. (2007). Methane oxyforming for synthesis gas production. Catal. Rev..

[33] Raju A.S.K., Park C.S., Norbeck J.M. (2009). Synthesis gas production using steam hydrogasification and steam reforming. Fuel Process. Technol..

[34] Lee J., Choi N.J., Lee H.K., Kim J., Lim S.Y., Kwon J.Y., Lee S.M., Moon S.E., Jong J.J., Yoo D.J. (2017). Low power consumption solid electrochemical-type micro CO_2_ gas sensor. Sens. Actuators B Chem..

[35] Fergus J.W. (2007). Materials for high temperature electrochemical NOx gas sensors. Sens. Actuators B Chem..

[36] Pang X., Shaw M.D., Gillot S., Lewis A.C. (2018). The impacts of water vapour and co-pollutants on the performance of electrochemical gas sensors used for air quality monitoring. Sens. Actuators B Chem..

[37] Serafini M., Mariani F., Gualandi I., Decataldo F., Possanzini L., Tessarolo M., Fraboni B., Tonelli D., Scavetta E. (2021). A wearable electrochemical gas sensor for ammonia detection. Sensors.

[38] Suresh M., Vasa N.J., Agarwal V., Chandapillai J. (2014). UV photo-ionization based asymmetric field differential ion mobility sensor for trace gas detection. Sens. Actuators B Chem..

[39] Pyo S., Lee K., Noh T., Jo E., Kim J. (2019). Sensitivity enhancement in photoionization detector using microelectrodes with integrated 1D nanostructures. Sens. Actuators B Chem..

[40] Zhu H., She J., Zhou M., Fan X. (2019). Rapid and sensitive detection of formaldehyde using porTable 2-dimensional gas chromatography equipped with photoionization detectors. Sens. Actuators B Chem..

[41] Li M.W.-H., Ghosh A., Sharma R., Zhu H., Fan X. (2021). Integrated microfluidic helium discharge photoionization detectors. Sens. Actuators B Chem..

[42] Vyas J.C., Katti V.R., Gupta S.K., Yakhmi J. (2006). V A non-invasive ultrasonic gas sensor for binary gas mixtures. Sens. Actuators B Chem..

[43] Minglei S., Xiang L., Changping Z., Jiahua Z. (2010). Gas concentration detection using ultrasonic based on wireless sensor networks. Proceedings of the 2nd International Conference on Information Science and Engineering.

[44] Costa M.M., Freire R.C.S., Villanueva J.M.M., Martins V.S.G. (2012). Concentration H 2 measurement and uncertainty analysis using ultrasonic transducer. Proceedings of the 2012 IEEE International Instrumentation and Measurement Technology Conference Proceedings.

[45] Ding X., Shi Y., Sun H., Ding X. (2021). A dual-frequency phase-difference method for ultrasonic hydrogen-concentration detection. Rev. Sci. Instrum..

[46] Chintoanu M., Ghita A., Aciu A., Pitl G., Costiug S., Cadar S., Ferenczi L., Cordos E. (2006). Methane and carbon monoxide gas detection system based on semiconductor sensor. Proceedings of the 2006 IEEE International Conference on Automation, Quality and Testing.

[47] Zhang J., Qin Z., Zeng D., Xie C. (2017). Metal-oxide-semiconductor based gas sensors: Screening, preparation, and integration. Phys. Chem. Chem. Phys..

[48] Li B., Zhou Q., Peng S., Liao Y. (2020). Recent advances of SnO_2_-based sensors for detecting volatile organic compounds. Front. Chem..

[49] Kang Y., Yu F., Zhang L., Wang W., Chen L., Li Y. (2021). Review of ZnO-based nanomaterials in gas sensors. Solid State Ionics.

[50] Tang H., Prasad K., Sanjines R., Levy F. (1995). TiO_2_ anatase thin films as gas sensors. Sens. Actuators B Chem..

[51] Wan Q., Li Q.H., Chen Y.J., Wang T.-H., He X.L., Li J.P., Lin C.L. (2004). Fabrication and ethanol sensing characteristics of ZnO nanowire gas sensors. Appl. Phys. Lett..

[52] Xu H., Liu X., Cui D., Li M., Jiang M. (2006). A novel method for improving the performance of ZnO gas sensors. Sens. Actuators B Chem..

[53] Karunagaran B., Uthirakumar P., Chung S.J., Velumani S., Suh E.-K. (2007). TiO_2_ thin film gas sensor for monitoring ammonia. Mater. Charact..

[54] Gupta S.K., Joshi A., Kaur M. (2010). Development of gas sensors using ZnO nanostructures. J. Chem. Sci..

[55] Zhang T., Nix M.B., Yoo B., Deshusses M.A., Myung N.V. (2006). Electrochemically functionalized single-walled carbon nanotube gas sensor. Electroanal. Int. J. Devoted Fundam. Pract. Asp. Electroanal..

[56] Kumar D., Chaturvedi P., Saho P., Jha P., Chouksey A., Lal M., Rawat J., Tandon R.P., Chaudhury P.K. (2017). Effect of single wall carbon nanotube networks on gas sensor response and detection limit. Sens. Actuators B Chem..

[57] Young S.J., Lin Z.D. (2018). Ethanol gas sensors based on multi-wall carbon nanotubes on oxidized Si substrate. Microsyst. Technol..

[58] Young S.-J., Liu Y.-H., Lin Z.-D., Ahmed K., Shiblee M.D.N.I., Romanuik S., Sekhar P.K., Thundat T., Nagahara L., Arya S. (2020). Multi-walled carbon nanotubes decorated with silver nanoparticles for acetone gas sensing at room temperature. J. Electrochem. Soc..

[59] Yoon H.J., Yang J.H., Zhou Z., Yang S.S., Cheng M.M.-C. (2011). Carbon dioxide gas sensor using a graphene sheet. Sens. Actuators B Chem..

[60] Chung M.G., Lee H.M., Kim T., Choi J.H., kyun Seo D., Yoo J.-B., Hong S.-H., Kang T.J., Kim Y.H. (2012). Highly sensitive NO_2_ gas sensor based on ozone treated graphene. Sens. Actuators B Chem..

[61] Lipatov A., Varezhnikov A., Wilson P., Sysoev V., Kolmakov A., Sinitskii A. (2013). Highly selective gas sensor arrays based on thermally reduced graphene oxide. Nanoscale.

[62] Yuan W., Shi G. (2013). Graphene-based gas sensors. J. Mater. Chem. A.

[63] Baek D.-H., Kim J. (2017). MoS2 gas sensor functionalized by Pd for the detection of hydrogen. Sens. Actuators B Chem..

[64] Kim Y., Kang S.-K., Oh N.-C., Lee H.-D., Lee S.-M., Park J., Kim H. (2019). Improved sensitivity in Schottky contacted two-dimensional MoS2 gas sensor. ACS Appl. Mater. Interfaces.

[65] Pham T., Li G., Bekyarova E., Itkis M.E., Mulchandani A. (2019). MoS2-based optoelectronic gas sensor with sub-parts-per-billion limit of NO_2_ gas detection. ACS Nano.

[66] Suh J.M., Shim Y.-S., Kwon K.C., Jeon J.-M., Lee T.H., Shokouhimehr M., Jang H.W. (2019). Pd-and Au-decorated MoS_2_ gas sensors for enhanced selectivity. Electron. Mater. Lett..

[67] Imai Y., Kimura Y., Niwano M. (2012). Organic hydrogen gas sensor with palladium-coated β-phase poly (vinylidene fluoride) thin films. Appl. Phys. Lett..

[68] Cavallari M.R., Izquierdo J.E.E., Braga G.S., Dirani E.A.T., Pereira-da-Silva M.A., Rodríguez E.F.G., Fonseca F.J. (2015). Enhanced sensitivity of gas sensor based on poly (3-hexylthiophene) thin-film transistors for disease diagnosis and environment monitoring. Sensors.

[69] Han S., Zhuang X., Shi W., Yang X., Li L., Yu J. (2016). Poly(3-hexylthiophene)/polystyrene (P3HT/PS) blends based organic field-effect transistor ammonia gas sensor. Sens. Actuators B Chem..

[70] Pasha A., Khasim S., Khan F.A., Dhananjaya N. (2019). Fabrication of gas sensor device using poly(3,4-ethylenedioxythiophene)-poly (styrenesulfonate)-doped reduced graphene oxide organic thin films for detection of ammonia gas at room temperature. Iran. Polym. J..

[71] Martinelli G., Carotta M.C., Ferroni M., Sadaoka Y., Traversa E. (1999). Screen-printed perovskite-type thick films as gas sensors for environmental monitoring. Sens. Actuators B Chem..

[72] Cerdà J., Arbiol J., Dezanneau G., Dıaz R., Morante J.R. (2002). Perovskite-type BaSnO_3_ powders for high temperature gas sensor applications. Sens. Actuators B Chem..

[73] Zhuang Y., Yuan W., Qian L., Chen S., Shi G. (2017). High-performance gas sensors based on a thiocyanate ion-doped organometal halide perovskite. Phys. Chem. Chem. Phys..

[74] Wang J., Ren Y., Liu H., Li Z., Liu X., Deng Y., Fang X. (2022). Ultrathin 2D NbWO_6_ Perovskite Semiconductor Based Gas Sensors with Ultrahigh Selectivity under Low Working Temperature. Adv. Mater..

[75] Zhao C., Fu J., Zhang Z., Xie E. (2013). Enhanced ethanol sensing performance of porous ultrathin NiO nanosheets with neck-connected networks. Rsc Adv..

[76] Zeng W., Liu Y., Chen G., Zhan H., Mei J., Luo N., He Z., Tang C. (2020). SnO–Sn_3_O_4_ heterostructural gas sensor with high response and selectivity to parts-per-billion-level NO 2 at low operating temperature. RSC Adv..

[77] Nakate U.T., Ahmad R., Patil P., Yu Y.T., Hahn Y.-B. (2020). Ultra thin NiO nanosheets for high performance hydrogen gas sensor device. Appl. Surf. Sci..

[78] Li Y., Chen N., Deng D., Xing X., Xiao X., Wang Y. (2017). Formaldehyde detection: SnO_2_ microspheres for formaldehyde gas sensor with high sensitivity, fast response/recovery and good selectivity. Sens. Actuators B Chem..

[79] Wu Z., Li Z., Li H., Sun M., Han S., Cai C., Shen W., Fu Y. (2019). Ultrafast response/recovery and high selectivity of the H_2_S gas sensor based on α-Fe_2_O_3_ nano-ellipsoids from one-step hydrothermal synthesis. ACS Appl. Mater. Interfaces.

[80] Zhang X., Sun J., Tang K., Wang H., Chen T., Jiang K., Zhou T., Quan H., Guo R. (2022). Ultralow detection limit and ultrafast response/recovery of the H_2_ gas sensor based on Pd-doped rGO/ZnO-SnO_2_ from hydrothermal synthesis. Microsystems Nanoeng..

[81] Lee J.-H., Kim J.-H., Kim J.-Y., Mirzaei A., Kim H.W., Kim S.S. (2019). ppb-Level selective hydrogen gas detection of Pd-functionalized In2O3-loaded ZnO nanofiber gas sensors. Sensors.

[82] Lee J.-H., Kim J.-Y., Kim J.-H., Kim S.S. (2019). Enhanced hydrogen detection in ppb-level by electrospun SnO_2_-loaded ZnO nanofibers. Sensors.

[83] Sui N., Zhang P., Zhou T., Zhang T. (2021). Selective ppb-level ozone gas sensor based on hierarchical branch-like In_2_O_3_ nanostructure. Sens. Actuators B Chem..

[84] Su P.-G., Zheng Y.-L. (2021). Room-temperature ppb-level SO_2_ gas sensors based on RGO/WO_3_ and MWCNTs/WO_3_ nanocomposites. Anal. Methods.

[85] Murali G., Reddeppa M., Seshendra Reddy C., Park S., Chandrakalavathi T., Kim M.-D., In I. (2020). Enhancing the charge carrier separation and transport via nitrogen-doped graphene quantum dot-TiO_2_ nanoplate hybrid structure for an efficient NO gas sensor. ACS Appl. Mater. Interfaces.

[86] He H., Guo J., Zhao J., Xu J., Zhao C., Gao Z., Song Y.-Y. (2022). Engineering CuMOF in TiO_2_ Nanochannels as Flexible Gas Sensor for High-Performance NO Detection at Room Temperature. ACS Sens..

[87] Wang J., Shen Y., Li X., Xia Y., Yang C. (2019). Synergistic effects of UV activation and surface oxygen vacancies on the room-temperature NO_2_ gas sensing performance of ZnO nanowires. Sens. Actuators B Chem..

[88] Kumar R., Goel N., Kumar M. (2017). UV-activated MoS2 based fast and reversible NO_2_ sensor at room temperature. ACS Sens..

[89] Drozdowska K., Rehman A., Krajewska A., Lioubtchenko D.V., Pavłov K., Rumyantsev S., Smulko J., Cywiński G. (2022). Effects of UV light irradiation on fluctuation enhanced gas sensing by carbon nanotube networks. Sens. Actuators B Chem..

[90] Li Y., Jin H., Sun G., Zhang B., Luo N., Lin L., Bala H., Cao J., Zhang Z., Wang Y. (2019). Synthesis of novel porous ZnO octahedrons and their improved UV-light activated formaldehyde-sensing performance by Au decoration. Phys. E Low-Dimens. Syst. Nanostruct..

[91] Chang H.-K., Ko D.-S., Cho D.-H., Kim S., Lee H.-N., Lee H.S., Kim H.-J., Park T.J., Park Y.M. (2021). Enhanced response of the photoactive gas sensor on formaldehyde using porous SnO_2_@ TiO_2_ heterostructure driven by gas-flow thermal evaporation and atomic layer deposition. Ceram. Int..

[92] Yang Y., Wu S., Cao Y., Li S., Xie T., Lin Y., Li Z. (2022). A highly efficient room-temperature formaldehyde gas sensor based on a Ni-doped ZnO hierarchical porous structure decorated with NiS illuminated by UV light. J. Alloys Compd..

[93] Seif A.M., Nikfarjam A., Hajghassem H. (2019). UV enhanced ammonia gas sensing properties of PANI/TiO_2_ core-shell nanofibers. Sens. Actuators B Chem..

[94] Zhou Y., Li X., Wang Y., Tai H., Guo Y. (2018). UV Illumination-enhanced molecular ammonia detection based on a ternary-reduced graphene oxide–titanium dioxide–Au composite film at room temperature. Anal. Chem..

[95] Yang Y., Yu S., Guo J., Zhang D. (2022). UV-enhanced highly sensitive ammonia sensing properties based on 2DPI/In2O3 heterostructure at room temperature. J. Alloys Compd..

[96] Chinh N.D., Hien T.T., Do Van L., Hieu N.M., Quang N.D., Lee S.-M., Kim C., Kim D. (2019). Adsorption/desorption kinetics of nitric oxide on zinc oxide nano film sensor enhanced by light irradiation and gold-nanoparticles decoration. Sens. Actuators B Chem..

[97] Xie Y., Yu S., Zhong Y., Zhang Q., Zhou Y. (2018). SnO_2_/graphene quantum dots composited photocatalyst for efficient nitric oxide oxidation under visible light. Appl. Surf. Sci..

[98] Geng Y., Chen D., Li N., Xu Q., Li H., He J., Lu J. (2021). Z-Scheme 2D/2D α-Fe_2_O_3_/g-C_3_N_4_ heterojunction for photocatalytic oxidation of nitric oxide. Appl. Catal. B Environ..

[99] Zhang B., Bao N., Wang T., Xu Y., Dong Y., Ni Y., Yu P., Wei Q., Wang J., Guo L. (2021). High-performance room temperature NO_2_ gas sensor based on visible light irradiated In_2_O_3_ nanowires. J. Alloys Compd..

[100] Chen P., Hu J., Yin M., Bai W., Chen X., Zhang Y. (2021). MoS2 nanoflowers decorated with Au nanoparticles for visible-light-enhanced gas sensing. ACS Appl. Nano Mater..

[101] Geng X., Lu P., Zhang C., Lahem D., Olivier M.-G., Debliquy M. (2019). Room-temperature NO_2_ gas sensors based on rGO@ ZnO_1-x_ composites: Experiments and molecular dynamics simulation. Sens. Actuators B Chem..

[102] Song Y., Zhang Y., Ma M., Ren J., Liu C., Tan J. (2020). Visible light-assisted formaldehyde sensor based on HoFeO_3_ nanoparticles with sub-ppm detection limit. Ceram. Int..

[103] Wang J., Deng H., Li X., Yang C., Xia Y. (2020). Visible-light photocatalysis enhanced room-temperature formaldehyde gas sensing by MoS_2_/rGO hybrids. Sens. Actuators B Chem..

[104] Huang J., Jiang D., Zhou J., Ye J., Sun Y., Li X., Geng Y., Wang J., Du Y., Qian Z. (2021). Visible light-activated room temperature NH_3_ sensor base on CuPc-loaded ZnO nanorods. Sens. Actuators B Chem..

[105] Shao X., Wang S., Hu L., Liu T., Wang X., Yin G., Zhou T., Rajan R., Jia F., Liu B. (2021). Improvement of Gas Sensing of Uniform Ag_3_PO_4_ Nanoparticles to NH_3_ under the Assistant of LED Lamp with Low Power Consumption at Room Temperature. ChemistrySelect.

[106] Lin Y.-T., Yu S.-Y., Zan H.-W., Yeh P.-H., Lu C.-J., Meng H.-F., Luo C.-W., Soppera O. (2019). Photo-assisted recovery in ammonia sensor based on organic vertical diode. Org. Electron..

